# H-Phosphonate Chemistry in the Synthesis of Electrically Neutral and Charged Antiviral and Anticancer Pronucleotides

**DOI:** 10.3389/fchem.2020.595738

**Published:** 2020-11-13

**Authors:** Adam Kraszewski, Michal Sobkowski, Jacek Stawinski

**Affiliations:** Institute of Bioorganic Chemistry, Polish Academy of Sciences, Poznań, Poland

**Keywords:** H-phosphonates, nucleotide analogs, pronucleotides, antiviral, anticancer

## Abstract

In this review a short account of our work on the synthesis and biological activity of electrically neutral and charged anti-HIV and anticancer pronucleotides, presented on the background of the contemporary research in this area, is given.

## Introduction

Nucleoside analogs were recognized early as antiviral compounds due to the rationally designed modifications of their natural structures that could interfere in viral life cycle and inhibit its replication. As flagship examples, one may list 9-(2-hydroxyethoxymethyl)guanine – an acyclo analog of guanosine [acyclovir, ACV] (Fyfe et al., 1978) and 3′-azido-3′-deoxythymidine (AZT) (Mitsuya et al., 1985) which were used to combat the herpes simplex (HSV) and human immunodeficiency (HIV) viruses, respectively. In malignancy, the recognition of thymidine biosynthesis was behind a concept of application of its analog, 5-fluoro-2′-deoxyuridine (FdU), as an inhibitor of cancer cell proliferation (Remy et al., 1962). Irrespective of therapeutic target, most (if not all) of the nucleoside analogs, must be phosphorylated into a nucleosid-5′-yl phosphate (for instance FdU → FdUMP) (Mukherjee and Heidelberger, 1962) or triphosphate by means of cellular (Furman et al., 1986) or viral (Fyfe et al., 1978) kinases to exert antiviral or anticancer activity. Thus, the biological potency of nucleoside prodrugs strongly depends on the efficiency of phosphorylation with cellular enzymes, which often is low and hampers their application in therapy [for recent comprehensive reviews, see Galmarini et al. (2002) and De Clercq (2005)].

To get around this weak point, efforts were focused onto delivery into the cell of already phosphorylated nucleoside analogs. Because of the known abortive uptake of nucleoside anionic mono-, di-, and triphosphates by cells (Remy et al., 1962) the studies involved electrically neutral nucleotide analogs, in which the phosphate residue usually bore lipophilic masking groups. These compounds, after entering the cell, underwent chemical or enzyme-assisted conversion into nucleotides with a free phosphate residue that only then could demonstrate the awaited biological activity. Thus, the above masked nucleotide analogs are progenitors of true nucleotide drugs and are called pronucleotides (Wagner et al., 2000; Mehellou, 2016; Mehellou et al., 2018).

Non-ionic pronucleotides are usually armed with lipophilic phosphate masking groups that are assumed to facilitate the penetration of the cell membrane and intracellular internalization of the compound. This indeed found positive verification in correlation of the lipophilicity (log*P*) and antiviral potency (low EC_50_ and high CC_50_)^1^. Within the last few decades, the electrical neutrality of pronucleotides and their high lipophilicity was commonly accepted as an obligatory requirement.

However, in the meantime several papers were published which described an application of anionic nucleotide analogs (Piantadosi et al., 1991; Krayevsky et al., 1992; Wagner et al., 1995; Pokrovsky et al., 2001; Schulz et al., 2014; Gollnest et al., 2016) that despite possessing a negative charge could enter the cell, and after the respective conversions effectively inhibited virus replication. These compounds were proven to act as pronucleotides and disclosed comparable or better antiviral activity in comparison to the parent nucleosides. Anionic nucleotide analogs were proven to be able to cross the cell membrane and act as pronucleotides with advantageous therapeutic indices. This weakened the aforementioned apparently inviolable rule of charge neutrality required for pronucleotides. High activity of anionic pronucleotides together with notably improved solubility in aqueous media facilitated their preclinical studies in various physiological milieux and made them very attractive candidates for antiviral therapy in the context of their administration (orally vs. systemic).

In typical approaches, pronucleotides are synthesized via phosphorylation of a nucleoside analog. Preparation of a respective phosphorylating agent was usually the weakest point of the whole synthetic route (Cahard et al., 2004; Pradere et al., 2014; Mehellou, 2016). This was not the case in the instance of nucleoside H-phosphates due to their unique chemical features and exceptional susceptibility to a variety of chemical transformations that made this class of compounds a particularly attractive alternative for many synthetic procedures. In this review we present applications for the H-phosphonate methodology for the preparation of antiviral and anticancer pronucleotides, including those that are difficult to obtain using other strategies.

## Uncharged Antiviral Pronucleotides

### H-Phosphonate Mono- and Diesters – Some Introductory Comments

In the synthesis of pronucleotides based on the *H-*phosphonate chemistry the first step is phosphonylation of therapeutic nucleoside analogs into the respective H*-*phosphonates, which are subsequently transformed into the desired P^V^ compounds. Successful realization of this strategy depends on synthetic access to the appropriate nucleoside H-phosphonates and the knowledge of their reactivity. Recently, the chemistry of H-phosphonates, including that of nucleoside H-phosphonates, was a subject of several comprehensive reviews from our laboratories (Stawinski, 1992a; Stawinski and Stromberg, 1993; Stawinski and Kraszewski, 2002a,b; Kraszewski and Stawinski, 2003, 2007; Sobkowski et al., 2015). Herein we recall the most convenient methods for the synthesis of H-phosphonate mono- and diesters, and present in detail those most synthetically useful in the preparation of pronucleotides. In principle, the introduction of a H-phosphonate function into alcohols could be done with phosphonic acid (H_3_PO_3_) activated with appropriate reagents or the use of reactive P(III) compounds. The most common activators of phosphonic acid are acyl chlorides but their effective applications require strictly controlled conditions (Stawinski and Thelin, 1990a,b). Among active phosphonylating reagents it is worth mentioning the pyro-H-phosphonate (Stawinski and Thelin, 1990b), salicylchlorophosphite (Marugg et al., 1986), PCl_3_/imidazole (Garegg et al., 1986), and PCl_3_/triazole (Froehler et al., 1986) systems. Separate types of phosphonylating reagents are reactive H-phosphonate diesters, e.g., bis(1,1,1,3,3,3-hexafluoro-2-propyl)phosphonate (Takaku et al., 1988) or bis(2,2,2-trifluoroethyl)phosphonate (Gibbs and Larsen, 1984), and diaryl H-phosphonate (Jankowska et al., 1994). Alkyl or aryl ester groups in these compounds are good leaving groups that may be substituted with one or two molecules of an alcohol. There are also 2-cyanoethyl (Venijaminova et al., 1994) or 9-fluorenemethyl H-phosphonates (Yang et al., 1995; Romanowska et al., 2009a) that are not reactive as such but may be activated, e.g., with pivaloyl chloride (PvCl), and effectively phosphonylate nucleoside OH function and produce the respective alkyl nucleoside H-phosphonate diesters. From these, upon treatment with tertiary amines in anhydrous conditions, the 2-cyanoethyl- or 9-fluorenemethyl groups can be removed quantitatively via β-elimination to furnish the respective H-phosphonate monoesters.

**Figure 1:**
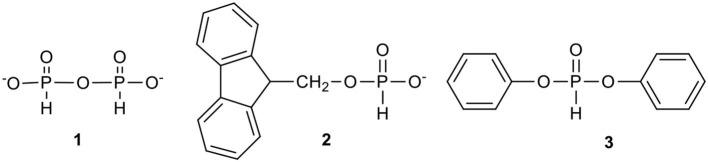
Convenient phosphonylating reagents in the synthesis of nucleoside H-phosphonates.

During our studies on nucleoside H-phosphonates and their analogs, the most synthetically useful, although for different reasons, were pyro-H-phosphonate **1**,9-florenemethyl H*-*phosphonate (Romanowska et al., 2009a) **2**, and diphenyl H*-*phosphonate (Jankowska et al., 1994) **3** (Figure 1).

Reagent **1** was found to be a useful but rather slow-acting monofunctional phosphonylating agent. It can be conveniently prepared in the reaction of phosphoric acid with a 0.5 molar equivalent of pivaloyl (or adamantanecarbonyl) chloride in pyridine. The reaction is very rapid (< 1 min) and the resulted pyro-H-phosphonate **1** is stable enough to be stored unchanged as a stock solution for several weeks. Usually, it is used in excess (at least 3 molar equiv.) to cleanly and efficiently produce the desired H-phosphonate monoester; however, the reaction requires several hours (recommended overnight) for completion.

**Scheme 1:**
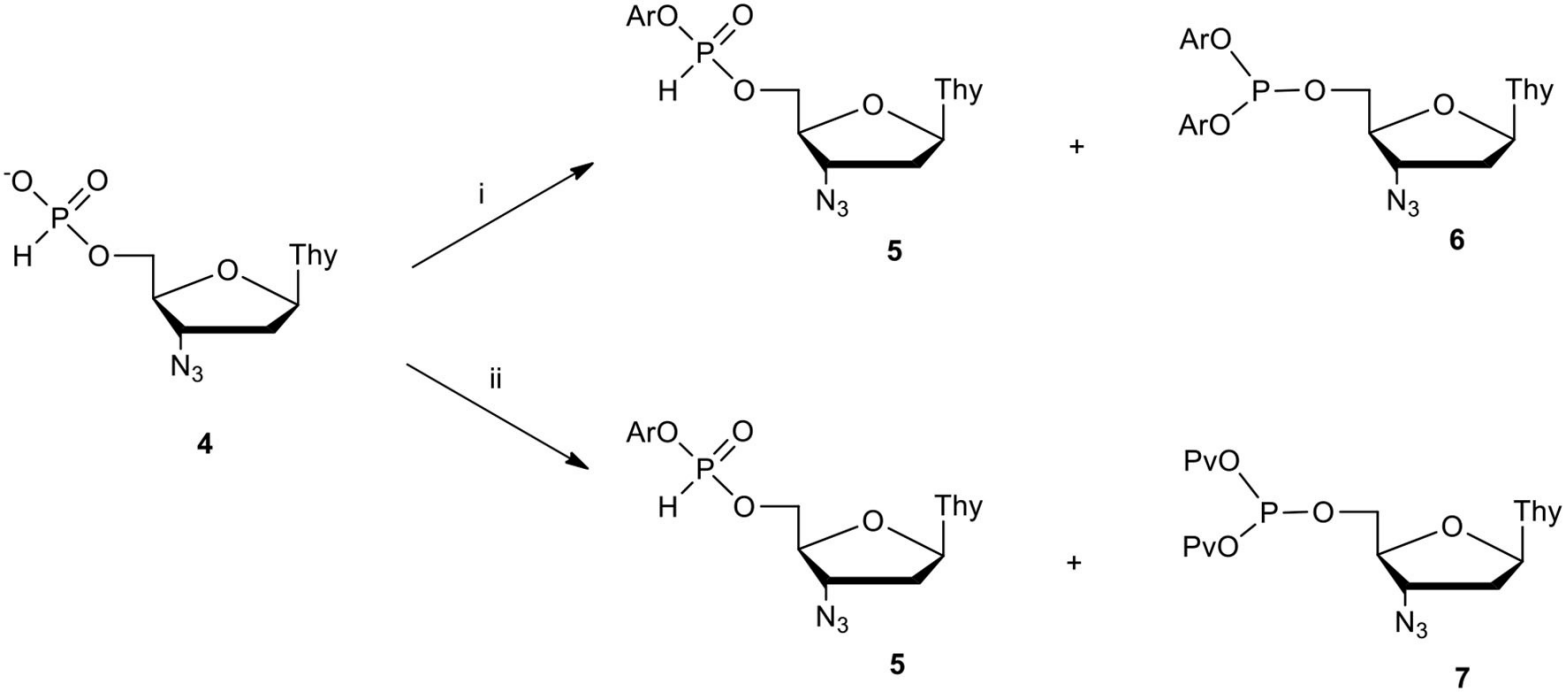
Attempted synthesis of aryl AZT H-phosphonate diesters. Conditions: ArOH (3 molar equiv.), condensing agent (C.A., 3 molar equiv.), pyridine; (i) C.A. = DPCP, (ii) C.A.= PvCl.

Pyro-H-phosphonate **1** was particularly useful in the synthesis of another type of phosphonylating reagent, 9-fluorenemethyl H-phosphonate **2** (Yang et al., 1995; Romanowska et al., 2009a) Typically, it is obtained in the reaction of 9-fluorenemethanol and an excess (3 molar equiv.) of pyro-H-phosphonate in pyridine. In an overnight reaction the whole 9-fluorenemethanol is converted into the respective H-phosphonate monoester, while the excess of the unreacted pyro-H-phosphonate is hydrolyzed with added water. The product **2**, due its high lipophilicity, may be isolated by means of simple solvent extraction (inorganic phosphoric acid is practically insoluble in most of organic solvents) to produce white crystals upon evaporation of the solvent. This phosphonylating reagent is particularly recommended for the phosphonylation of polar nucleosides (or other polar compounds) and their analogs since its lipophilic handle significantly facilitates the isolation of the product, 9-fluorenemethylene nucleoside H-phosphonate diester. The last reagent, when treated with tertiary amines (for instance triethylamine), undergoes rapid β-elimination and unleashes 9-methylene-9*H*-fluorene and nucleoside H-phosphonate and could be isolated by a simple extraction method as a solid product of high purity (> 99%). This approach was successfully used for the preparation of several 2′,3′-dideoxynucleoside H-phosphonates, including AZT *H-*phosphonate **4** (cf. Scheme 1) (Romanowska et al., 2009a).

The third phosphonylating reagent commonly used in our laboratories is diphenyl H-phosphonate (DPHP) **3**, a reagent in which the electron withdrawing effect of both the phenyl groups makes the phosphorus center sufficiently electrophilic to effect a rapid transesterification with alcohols, including nucleosides (Jankowska et al., 1994). DPHP is a low-cost, easily accessible, commercial reagent that when used in reasonable excess (3–7 molar equiv.), ensures a quantitative and fast (< 15 min.) phosphonylation of nucleosides under very mild conditions, giving access to compounds that are otherwise difficult to obtain. The second phenyl group may be easily cleaved by hydrolysis or used for the introduction of another ester group. An additional advantage of using DPHP is that protection of the amino functions in the nucleobases (Ade, Cyt, Gua) is not required (Wada et al., 1999). The three phosphonylating agents **1**–**3** satisfied most of our recent needs and their choice depended on type of nucleosidic substrate used for studies.

### Anti-HIV Diaryl Nucleoside Phosphotriesters

Considering the concepts of functioning antiviral pronucleotides, so far there are three ways they can be converted into the respective biologically active nucleotides. The first one employs cellular enzymes that remove phosphate masking groups as in *S*-acyl-2-thioethyl (SATE) pronucleotides (Ergon et al., 1998; Peyrottes et al., 2004), the second one uses chemical transformations exclusively like vide cyclo-saligenyl (cycloSal) pronucleotides (Meier et al., 1997), and the third one employs both enzymatic and chemical steps, as in McGuigan's ProTides (McGuigan et al., 2013), in which the first step is hydrolysis of the carboxyl ester by the cellular carboxyl esterase, followed by intramolecular substitution of the phosphoester aryl group and final hydrolysis of the P-N bond also by cellular phosphoamidases. The last pathway seems to be the most complex but it works perfectly well. We chose a variant of the third type of pronucleotides in which the phosphate residue was decorated with two aryl groups (Romanowska et al., 2009b). The rationale behind our choice of a two aryls phosphate masking system was as follows: (i) the first step of transformation should be independent of cellular enzymes, (ii) the kinetics of the chemical hydrolysis of the first aryl phosphoester should be controlled by electronic properties of the aryl groups, and (iii) the resulting chemically stable aryl phosphodiester should be easily cleaved by intracellular phosphoesterases. In the light of simple access to a variety of phenols it seems that the first two criteria should be relatively easy to fulfill. It could be presumed that the third one should not pose problems keeping in mind that 4-nitrophenyl phosphoester is a standard substrate in assays for phosphoestrase activity and it might be expected that other aryls will also be hydrolyzed relatively easily.

In our initial strategy of the synthesis of target diaryl pronucleotides we considered several variants, of which the first seemed to be the most simple, that assumed the formation of aryl nucleoside H-phosphonates followed by their conversion into the final diaryl nucleoside phosphates. Unfortunately, when phenols were coupled with nucleoside H-phosphonate **4** in pyridine, the awaited aryl nucleoside H-phosphonate diester **5** were invariably contaminated with various amounts of by-products, the type and amount of which depended on the condensing agent used and – interestingly – the type of the phenol used. In the case of arylols of low acidity (e.g., unsubstituted phenol or toluol), diester **5** was the predominant product together with diaryl nucleoside phosphite **6** when diphenyl chlorophosphate (DPCP) was used as a condensing agent (C.A.) or bis(pivaloyl) phosphite **7** in the instance of PvCl (Scheme 1) (Cieslak et al., 1996).

**Scheme 2:**
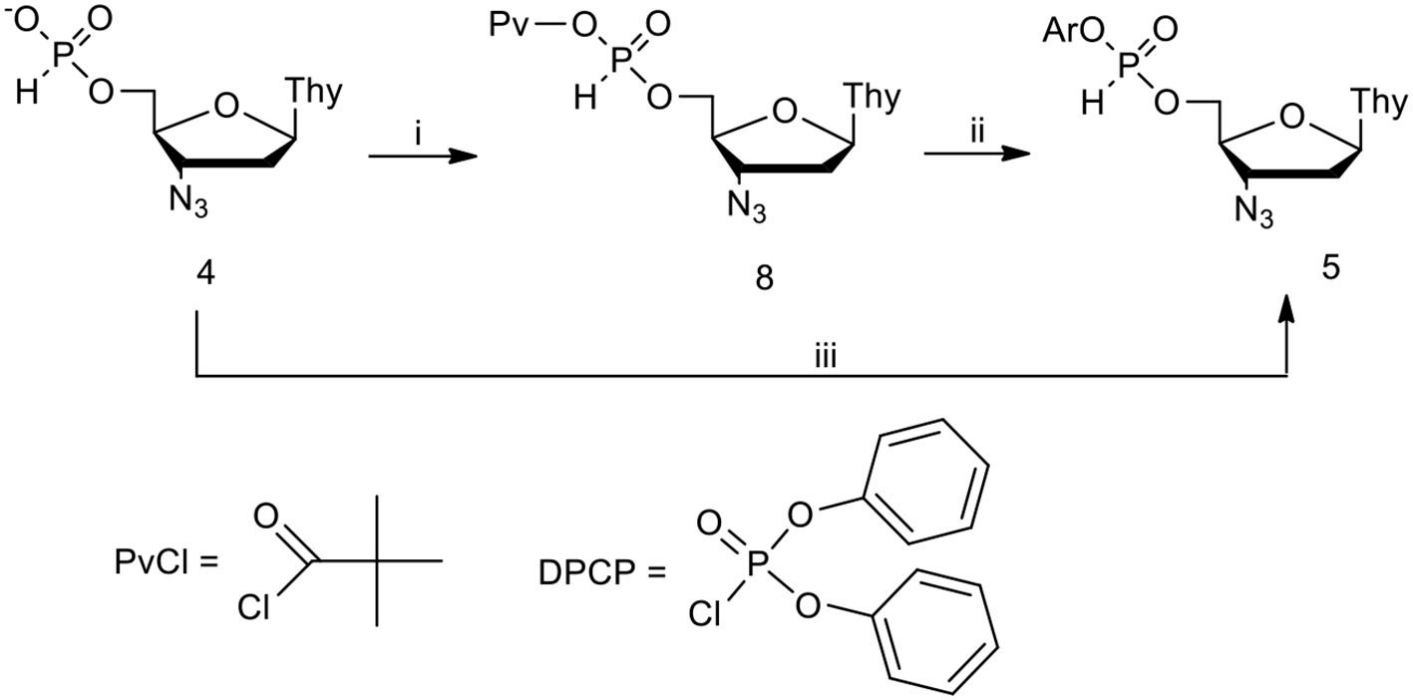
Formation of aryl AZT H-phosphonate diesters in CH_2_Cl_2_/pyridine 9: 1 (v/v). (i) PvCl, 1.2 molar equiv.; (ii) ArOH 1.5 molar equiv.; (iii) DPCP 1.2 molar equiv., ArOH 1.5 molar equiv.

For phenols of higher acidity, the content of tervalent phosphites of type **6** or **7** increased. Decreasing the amount of the coupling agent did not improve yields of **5** and in each case the presence of the unreacted H-phosphonate **4** was observed. Additional studies on the activation of monoester **4** with pivaloyl chloride in pyridine in the absence of a nucleophile revealed that when the stoichiometric ratio of reagents was used, the reaction mixture contained bis(pivaloyl) phosphite **7** (δ_P_ 123 ppm, ^31^P NMR) and H-phosphonate **4** (δ_P_ ca 2 ppm, ^31^P NMR) in a ca. 1:1 ratio. The addition of the next portion of PvCl caused the formation of additional amounts of the tervalent product **7**, while the expected pivaloyl – H-phosphonate mixed anhydride of type **8** (δ_P_ ca 5 ppm, ^31^P NMR; Scheme 2) was not observed. All above experiments strongly suggested that the activation of the phosphorus center in H-phosphonates with electron withdrawing substituents, as in mixed anhydride of type **8** (for instance, acyl chlorides or strongly acidic phenols) in basic solutions (e.g., pyridine), triggered a rearrangement of the active intermediate products of type **5** or **8** toward tervalent congeners of type **6** or **7**, respectively.

This hypothesis was verified positively in reactions in which all reagents were used close to the stoichiometric ratio and were carried out in neutral solvents in the presence of controlled excess (5–12 molar equiv.) of pyridine. Under such weak basic conditions, the reaction with pivaloyl chloride furnished pivaloyl H-phosphonate mixed anhydride **8** (ca. 5 ppm) as the only species observed (^31^P NMR) (Cieslak et al., 1996).

The addition of phenols of weak or moderate acidity cleanly and quantitatively produced the awaited aryl nucleoside H-phosphonate of type **5**. More acidic phenols reacted with the pre-formed mixed anhydride **8** toward a mixture of aryl nucleoside H-phosphonate **5** and an “unreacted” **8**, in a ratio depending on p*K*_a_ and the amount of the phenol used. The last reaction strongly indicated an existence of equilibrium between the two reactive compounds, mixed anhydride **8** and diester **5**. This was not the case when DPCP was used as the coupling agent (most probably, the putative mixed phosphate – H-phosphonate mixed anhydride was significantly more reactive than its carboxy congener **8**). In these reactions, H-phosphonate diesters **5** were the sole products for phenols of a wide p*K*_a_ range of (7.14–10.19, for 4-nitrophenol and 4-methylphenol, respectively) (Perrin, 1965; Cieslak et al., 1999).

**Scheme 3:**
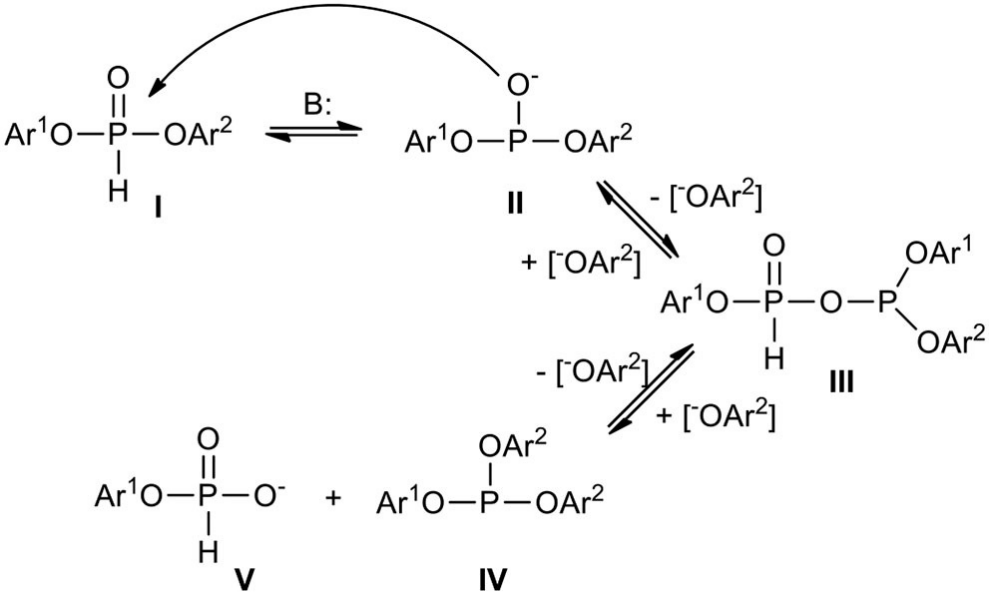
Scheme of structural phosphonate-phosphite disproportionation of an exemplary diaryl H-phosphonate.

The seemingly ambiguous results shown above support the hypothesis that H-phosphonate diesters or diester-like compounds may undergo phosphonate-phosphite structural disproportionation (Kers et al., 1996) (Scheme 3), the kinetics of which strongly depend on the electron releasing or attracting properties of substituents at the phosphorus atom. It is well-known that H-phosphonates **I** are in equilibrium with their tervalent tautomers **II** which can react one with another with formation of H-phosphonate-phosphites **III** and release of aryloxy anion [Ar^2^O^−^] that in turn in reaction with **III** produces a mixture of phosphite triester **IV** and H-phosphonate monoester **V**. It seems that such a transformation is an inherent feature of H-phosphonate diesters bearing electron withdrawing substituents (e.g., aryls with deactivated aromatic ring, acyls, and similar) that manifest particularly under basic conditions. In general, the more electron withdrawing substituents in **I** and more basic condition, the more the equilibrium is shifted toward **IV** and **V**. It is worth noting at this point that simple dialkyl H-phosphonates also undergo disproportionation toward alkyl H-phosphonate monoesters and dialkyl alkylphophonates but in much harsher conditions. These reactions are governed by alkylation/dealkylation rather than by substitution (Pelchowicz et al., 1961; Zimmerer and Laughlin, 1962).

**Scheme 4:**
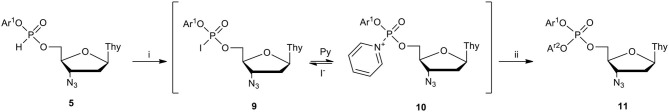
Synthesis of diaryl nucleoside phosphate via oxidative coupling on the example of AZT H-phosphonate **5**. (i) I_2_, 2 molar equiv. in pyridine (1 mM/mL); (ii) Ar^2^OH, 5 molar equiv.

The development of synthetic procedures for the efficient formation of aryl nucleoside H-phosphonates of type **5** was a very important development because these compounds are very difficult to isolate due to their high reactivity (Cieslak et al., 1999) and thus, have to be prepared *in situ*. After probing several synthetic variants for the introduction of the second aryl group into diester **5**, we chose an oxidative coupling approach [for reviews, see Stawinski (1992a), Stawinski and Kraszewski (2002b), Kraszewski and Stawinski (2007)] with iodine as an oxidant. In these reactions, the generated, highly reactive species, phosphoriodidates **9** and pyridinium-1-ylphosphonate **10** (McCombie and Stacey, 1945; Garegg et al., 1987; Stromberg and Stawinski, 1987), reacted with a chosen phenol (Ar^2^OH) yielding the desired triester **11** (Romanowska et al., 2009b) (Scheme 4). In practice, Ar^2^OH was added prior to I_2_ to minimize the presence of adventitious water when the most reactive species **9** and/or **10** were generated. In the synthesis of unsymmetrical diaryl nucleoside phosphates **11**, the order of introduction of the aryl groups appeared to be critical for a smooth course of the reaction. Thus, a less acidic phenol (Ar^1^OH) should be used for the preparation of H-phosphonate **5**, while the Ar^2^OH used in the oxidative coupling step should be more acidic. If a reverse order of addition of phenols were to be used, the preformed H-phosphonate **5** would suffer significant transesterification prior to the oxidative coupling and the formation of symmetrical diaryl nucleoside phosphotriesters would compromise the whole procedure. Later, it was found that using pyridinols as arylols was particularly beneficial because the presence of pyridinium moieties in the pronucleotides enhanced their solubility in aqueous media, while keeping them sufficiently lipophilic to facilitate their membrane permeability.

Compounds to be used as pronucleotides have to be stable enough to survive the physiological environment at the entrance to the target cells. Typically, the first step to gain some knowledge on stability and to recognize possible routes of decomposition *in vivo* is by the incubation of a candidate pronucleotide in a standard cell culture media [RPMI/FCS (10%)]. This makes it possible to determine which compound(s) cells are exposed and which of them might be responsible for biological activity. When diaryl pronucleotides of type **11** were examined in the above context it turned out that their stability correlated well with p*K*_a_ of the pair of the aryls masking the phosphate residue. Their t_1/2_ varied significantly (in between 3 and 80 h) and were shortest for those carrying more acidic arylols (e.g., 173 min for Ar^1^ = 4-cyanophenyl, Ar^2^ = pyridyn-3-yl) (Romanowska et al., 2009b). From this we have learned that by changing the aryl groups in pronucleotides **11** it was possible to control the stability of these compounds in biological media.

**Scheme 5:**
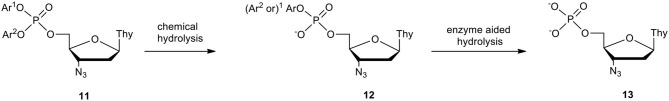
Pronucleotide metabolism in cell culture media (RPMI/FCS 10%) exemplified on AZT bis-aryl phosphotriester **11**.

Enzymes catalyzing a hydrolytic cleavage of phosphotriesters are very rare (Shim et al., 1998) and phosphotriesterases that might be involved in the metabolism of nucleotide phosphotriesters are unknown. Since phosphate triesters are quite prone to hydrolysis, the first step of degradation of nucleotide phosphotriesters **11** in cell culture media [RPMI/FCS 9: 1 (v/v)] is assumed to have a purely chemical character (Scheme 5). In contrast, phosphodiesters are very resistant to hydrolysis, while phosphodiesterases, commonly present in cells, cleave them rapidly. Thus, aryl nucleoside phosphodiesters of type **12**, which can survive in neat RPMI for many days, were hydrolyzed within several hours in fetal calf serum (FCS) containing phosphoesterases supplemented with RPMI and produced awaited nucleoside phosphates **13**. The above cell-free experiments delivered precise data which were helpful to evaluate the chances for the pronucleotide to act as such or whether the biological effect would occur with its metabolite preformed in cell culture media.

A dozen of anti-HIV diaryl pronucleotides were synthesized using the described above approach including derivatives of 3′-deoxy-3′-azidothymidine (AZT), 2′,3′-dideoxyadenosine (ddA), and 2′,3′-dideoxyuridine (ddU), and their anti-HIV potency (EC_50_) and cytoxicity (CC_50_) against MT-4 cells were evaluated (Romanowska et al., 2009b). Several of them showed advantageous antiviral parameters in comparison to the parent nucleoside AZT (EC_50_ = 0.01 μM) but were much less toxic. Low cytotoxicity appeared to be a common feature of most pronucleotides studied by us and others, making these compounds particularly attractive candidates for antiviral therapy.

Since nucleoside phosphates can decompose in the cell into nucleosides and inorganic phosphates, there is a constant dilemma of pronucleotidic vs. pronucleosidic character of the investigated compounds. This can be solved to some extent by analyzing antiviral properties of diaryl ddU pronucleotide, since non-phosphorylated ddU is completely inactive (it is not a substrate for cellular kinases and cannot be converted into respective mono-, di- and finally, triphosphate – the true inhibitor of viral reverse transcriptase). We found that diaryl ddU phosphate of type **11** (Ar^1^ = phenyl, Ar^2^ = pyridin-4-yl) exhibited rather low (EC_50_ 3.0 μM) but we observed a concentration dependent anti-HIV activity suggesting that it was converted into a nucleotide within the cells. Thus, we confirmed, that diaryl dideoxynucleoside phosphates at least in part can act as pronucleotides.

Studies on diaryl nucleoside phosphates of type **11** proved that by choosing proper aryl masking groups of the phosphate residue it is possible to optimize their pronucleotidic features, i.e., lipophilicity, stability in biological media, enzymatic substrate properties, and increase their antiviral or anticancer activity.

### Anti-HIV Pronucleotides Bearing the P-N Bond

In the course of our studies on antiviral pronucleotides, we turned our attention to phosphorous derivatives containing the P-N bond because this type of compound could significantly widen the variety of potential antiviral drugs. Having easy access to nucleoside H-phosphonates of type **4**, we started the development of efficient methods for their reactions with amines. It was found that the formation of *N*-alkyl or *N*-aryl nucleoside H-phosphonamidates strongly depended on several factors, mainly the type of amine, and the condensing agent used, and the reaction milieu (Sobkowska et al., 1997; Romanowska et al., 2011, 2019). Standard coupling of nucleoside H-phosphonate with unhindered primary or secondary amines in pyridine produced a multi-product mixture containing unreacted monoesters of type **4** and a number of tervalent species, most likely products of phosphonate-phosphite disproportionation (vide supra) and their aminolysis. Much better results were obtained when neat pyridine was substituted with a dichloromethane (DCM)/pyridine 9:1 (v/v) mixture and H-phosphonate **4** was preactivated with PvCl (toward the mixed anhydride **8**, Scheme 2) prior to the addition of an amine. Under such conditions, a significantly simpler product composition was obtained and in the case of unhindered aliphatic amines it contained the awaited *N*-alkyl H-phosphonamidate accompanied by minor amounts of monoester **4** (usually in ratio 4: 1). Apparently, amines attacked both the carbonyl carbon and phosphorus electrophilic centers of the mixed anhydride **8**. Under the above conditions hindered amines, e.g., *N,N*-diisopropylamine, invariably produced an equimolar mixture of H-phosphonate **4** and nucleoside bis(pivaloyl)phosphite of type **7** (Scheme 1) as products of exclusive phosphonate-phosphite disproportionation, with no traces of the expected H-phosphonamidates of type **17**.

**Scheme 6:**
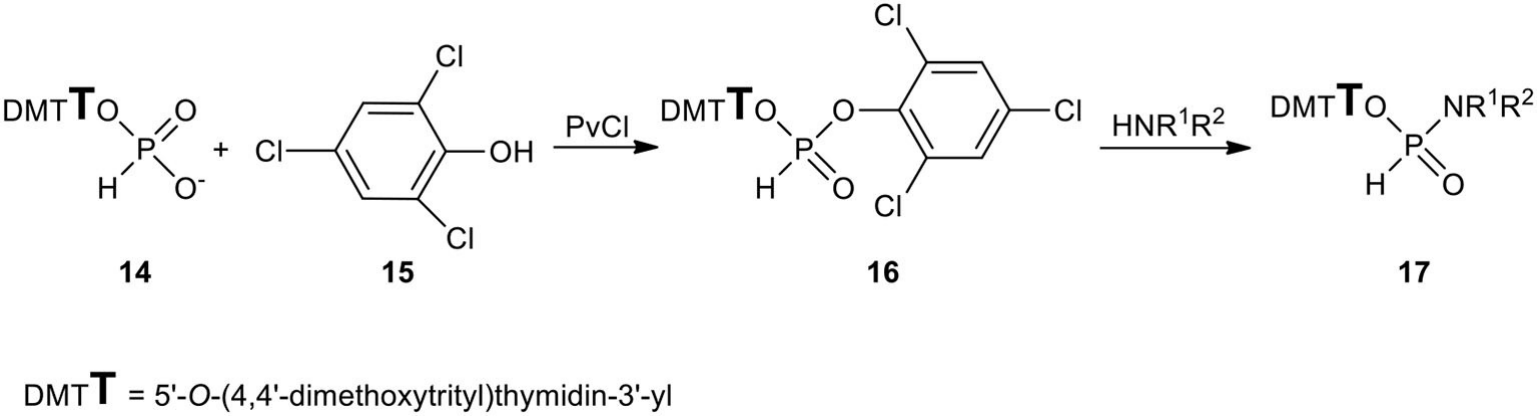
Scheme for the synthesis of *N*-alkyl-H-phosphonamidates of type **17**.

All the above synthetic inconveniences were circumvented by using the intermediacy of 2,4,6-trichlorophenyl nucleoside H-phosphonate **16** that was formed quantitatively and reacted smoothly with most of the primary and secondary aliphatic amines toward the desired nucleoside *N*-alkyl H-phosphonamidates (Scheme 6). However here again, the exception were hindered amines that triggered the rapid phosphonate-phosphite disproportionation instead of acting as nucleophilic reactants (Kers et al., 1996). Finally, H-phosphonamidates **17** bearing bulky *N*-alkyl substituents were obtained in smooth but slow (several hours) reactions of the respective amine and nucleoside H-phosphonate (Sobkowska et al., 1997) aided by 2-chloro-5,5-dimethyl-1,3,2-dioxaphosphinane 2-oxide (neopentylidene chlorophosphate) (McConnell and Coover, 1959).

**Scheme 7:**
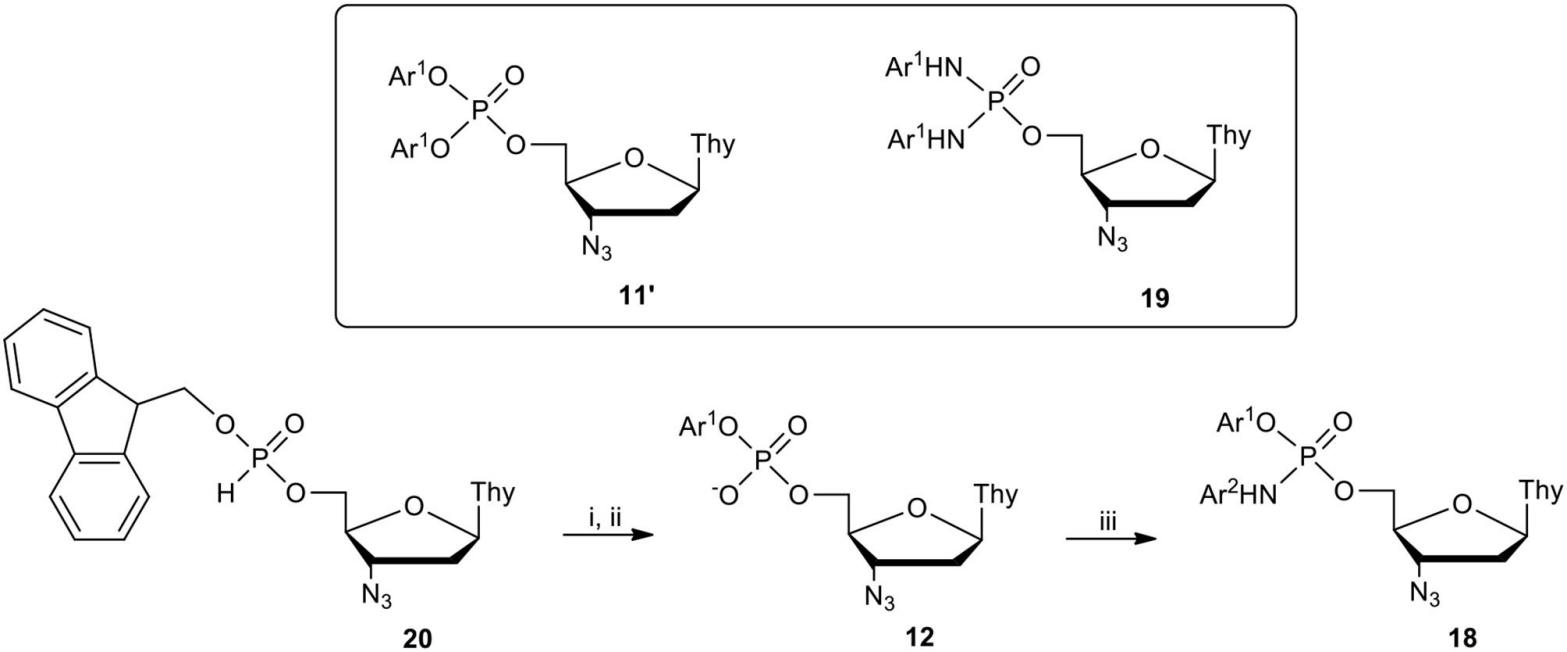
Scheme of the synthesis of aryl nucleoside (*N*-aryl)phosphoramidates via aryl nucleoside phosphodeisters on the example of AZT derivatives. (i) I_2_, Ar^1^OH, 1.5 molar equiv. both; (ii) CH_3_CN/Et_3_N 2: 1 (v/v), 10 mL/1 mmol, 20 min.; (iii) Ar^2^NH_2_ 1.5 molar equiv., TPSCl 3 molar equiv. in CH_2_Cl_2_/MeIm 9: 1 (v/v), 10 mL/1 mmol, 60 min.

Unfortunately, nucleoside *N*-alkyl H-phosphonamidates **17** appeared to be quite prone to hydrolysis and required special caution during work. Moreover, they reacted reluctantly with electrophiles and for example, their attempted oxidation with the I_2_/pyridine/water system (which is an extremely rapid reaction for H-phosphonate diesters) proceeded very slowly and produced a P-N bond hydrolytic scission and the recovery of the nucleoside H-phosphonate monoester **4**. Because of these difficulties and the rather low chances of obtaining the prospective pronucleotides from *N*-alkyl H-phosphonamidates **17**, we abandoned this strategy and started an exploration into aryl nucleoside (*N*-aryl)phosphoramidates of type **18** (Scheme 7) that were poorly recognized as potential antiviral or anticancer therapeutics.

Phosphoester and phosphoramidate bonds have different chemical natures and their presence in one molecule might be a decoy for new concepts of pronucleotides with structures fine-tuned to optimize their antiviral or anticancer activity. Because of reasons similar to diaryl pronucleotides (vide supra), our studies were limited to nucleoside aryl (*N*-aryl)phosphoramidates bearing mainly pyridinyl masking groups. It is worth noting that while the presence of the P-N bond beside of the P-O bond likely enriched the potential biological applications of phosphoramidates **18**, it is also likely that their synthesis may be more challenging. Keeping in mind the benefits of a one-pot approach in the synthesis of diaryl triester pronucleotides of type **11** we were eager to apply the same methodology for iso-structural aryl nucleoside (*N*-aryl)phosphoramidates of type **18** (Scheme 7). Since the key substrates were nucleoside *H-*phosphonates, our synthetic strategy was to form a new phosphoester bond as the first step (in a condensing agent-promoted reaction with a phenol), followed by the introduction of a phosphoramidate bond (iodine promoted oxidative coupling with an arylamine). This order of reactions was in line with the known high reactivity of alcohols toward activated H-phosphonates and amines toward P^V^-halogen compounds (Stawinski et al., 1992; Nilsson and Stawinski, 2004; Nilsson et al., 2005).

Unfortunately this synthetic route appeared to only run well for a limited number of phenol-aromatic amine pairs and its efficacy strongly depended on the p*K*_a_ of the phenol and arylamine used. Thus, in many cases the yield of the expected phosphoramidate diester **18** was reduced by the formation of symmetrical diaryl phosphotriester **11′** or analogous bis(*N*-aryl)phosphordiamidate **19** (Scheme 7, inset) due to unexpectedly rapid ester-to-amid exchange and the competitive reactions of the two nucleophiles present in a one-pot during the oxidative condensation step (Romanowska et al., 2019).

Surprisingly, contrary to our initial strategy, in the most effective method, the P-O-aryl bond was formed via oxidative coupling, while the P-N-aryl bond was formed via condensation (Scheme 7). Moreover, instead of using nucleoside *H-*phosphonate monoester of type **4** as a substrate, the procedure started with phosphonylation of a nucleoside with (9*H*-fluroren-9-yl)methyl *H-*phosphonate **2** (Figure 1) to produce a protected *H-*phosphonate diester **20** (Romanowska et al., 2009a), which was oxidatively coupled with a respective phenol to a phosphotriester (structure not shown).

A subsequent removal of the 9-methylene-9*H*-fluorenyl protecting group with triethylamnie via β-elimination furnished aryl nucleoside phosphate **12** that after coupling aided with 2,4,6-triisopropylbenzenesulfonyl chloride (TPSCl)/*N*-methylimidazole (Efimov et al., 1985) (MeIm) gave the final product, amidoester **18**. This approach, although time consuming and less atom economic than the other examined method, can be recommended as the general method for the preparation of the aryl nucleoside (*N*-aryl)phosphoramidate of type **18**. It should be mentioned that phosphoramidates containing pyridin-4-yl ester group were unstable during workup and purification and their biological potency could not be evaluated (Romanowska et al., 2019).

**Scheme 8:**
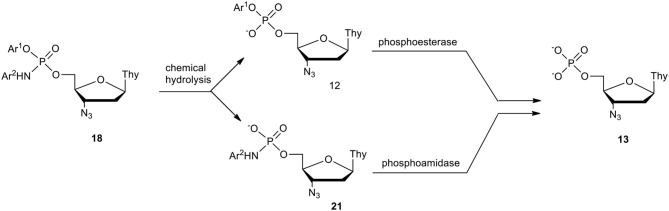
Plausible pathways of decomposition of aryl nucleoside (*N*-aryl)phosphoramidate **18** in RPMI/FCS on the example of AZT derivatives.

In our studies on degradation paths of pronucleotides **18** in cell culture media (RPMI), we expected that in the chemical hydrolysis of the phosphoramidate diesters **18**, the departure of the acidic phenol over cleavage of the P-N bond should be preferential (Scheme 8). Surprisingly, this was only the case for derivatives containing an aniline moiety, while for the other Ar^1^-Ar^2^ combinations the hydrolysis led to a mixture of phosphodiesters of type **12** and phosphoramidates of type **21** in various proportions. However, since cells contain both phosphoesterases (Purich and Allison, 2002) and phosphoamidases (Abraham et al., 1996; Wagner et al., 1999; Guranowski et al., 2010a,b), we expected that phosphoesters **12** and phosphoramidates **21** will be hydrolyzed toward AZT phosphate **13**, the precursor of AZTTP, the true inhibitor of HIV reverse transcriptase (HIV-RT). Additionally, as described by Adelfinskaya and Herdewijn (2007) (Adelfinskaya et al., 2007; Zlatev et al., 2009; De et al., 2016) it is possible that phosphoramidates of type **21** as such can be substrates for polymerases (including HIV reverse transcriptase) and in this way also inhibit viral cDNA synthesis.

The half time for decomposition of the examined aryl nucleoside (*N*-aryl)phosphoramidates **18** in cell culture media varied (25 h−8 d) and their stability mostly depended on the kind of phosphoester than on the phosphoramidate group (Romanowska et al., 2019). In several instances, the anti-HIV potency parameters (EC_50_, CC_50_ and SI) were encouraging, although in none of the cases, were they notably advantageous over the parent nucleoside. The improvement of this type of pronucleotide calls for further studies.

A pronucleotidic mode of action of aryl nucleoside (*N*-aryl)phosphoramidates **18** was checked by the preparation and examination of ddU derivatives that also in this instance disclosed a pronounced anti-HIV activity (Romanowska et al., 2019). The attempted correlation of the aryl masking groups in compounds **18**, their stability in cell culture media, metabolic paths (phosphodiesters vs. phosphoramidates), with their anti-HIV activity, gave no clear-cut result, although certain trends were observed. For instance, the highest antiviral potency was found for compounds that were hydrolyzed fastest (the shortest t_1/2_). On the other hand, phenyl AZT [*N-*pyrid-2(or 3)-yl]phosphoramidates that were very stable (t_1/2_ 7 days and 16 days, respectively), showed lower anti-HIV activity but had low cytotoxicity that resulted in higher selectivity indices. At present we can only speculate that due to the degradation pathways of pronucleotides of type **18** (Scheme 8), their antiviral potency might strongly depend on the contribution of metabolites of type **21**, which were found highly active against HIV (Romanowska et al., 2011) (vide infra).

### Anti-HIV Pronucleotides With a P-C Bond

#### Dinucleoside Phosphonate-Phosphates

**Scheme 9:**
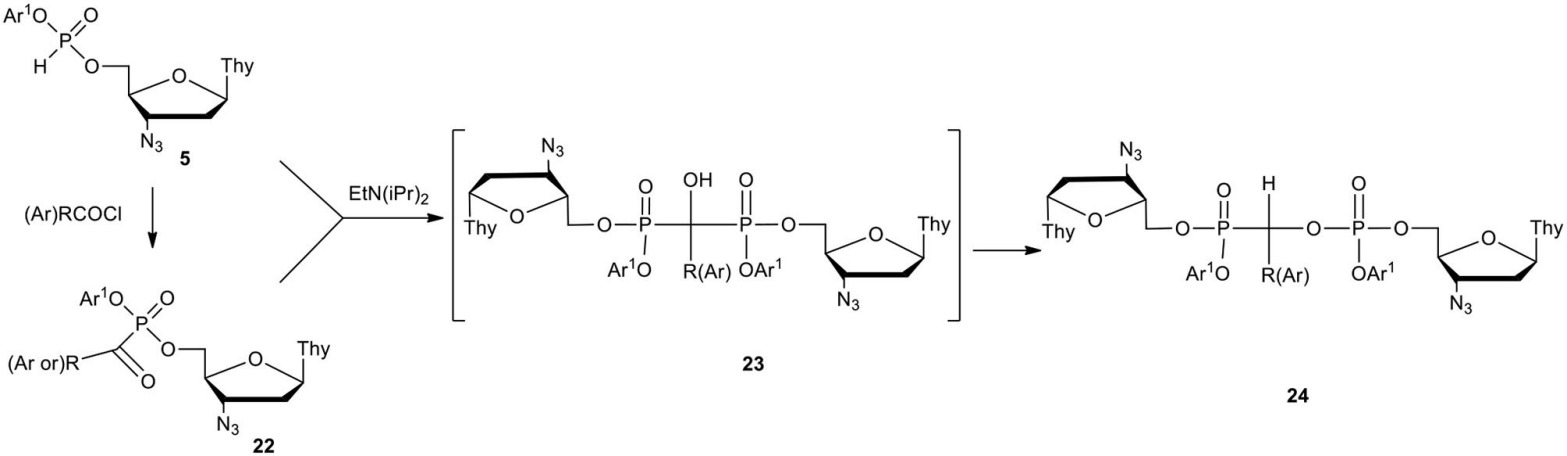
Scheme for the synthesis of fully masked dinucleoside phosphonate-phosphates **24** on the example of AZT derivatives.

Phosphonate-phosphates are a class of compounds of type **24** (Scheme 9) in which a C-phosphonate carbon atom is attached to the phosphate residue. It is not surprising that the presence of two different phosphorus centers in one molecule stimulated the new concept of phosphonate-phosphates as nucleotide prodrugs. It could be assumed that phosphonate-phosphates bearing biologically active nucleoside moieties on the P-C and P^V^ parts would undergo decomposition with the formation of at least two active compounds, namely, nucleoside phosphonate and nucleotide [for a review, see (Hecker and Erion, 2008; Balzarini et al., 2019]. Alkyl phosphonate-phosphates were obtained by the rearrangement of the respective *gem*-diphosphonates (Nicholson and Vaughn, 1971; Gancarz and Gancarz, 1993) of type **23** and found therapeutic applications (Nguyen et al., 1987; Turhanen et al., 2000). As the rearrangement requires rather harsh conditions (Fitch and Moedritzer, 1962), alkyl phosphonate-phosphates have not been of interest in nucleotide chemistry. In contrast, aryl nucleoside H-phosphonates **5** easily underwent P-acylation with acyl chlorides toward α-ketophosphonate **22** that in the presence of an added tertiary base reacted rapidly with the remaining compound **5**, forming a dinucleoside diaryl phosphonate-phosphate of type **24** (Scheme 9) (Szymczak et al., 2003).

On the mechanistic ground, the formation of phosphonate-phosphate **24** has to proceed with the intermediacy of α-hydroxy bisphosphonate of type **23** that, unlike the alkyl analogs, rapidly undergoes phosphonate-phosphate rearrangement into unsymmetrical compound of type **24**, and could not be isolated or even observed with commonly used analytical methods (e.g., TLC or ^31^P NMR). In practice, when both P-C and P^V^ aryl esters are identical, an equimolar mixture of aryl H-phosphonate **5** and P-acyl aryl ester **22** can be prepared easily by partial acylation of **5** which upon the addition of a tertiary base, smoothly produced the awaited phosphonate-phosphate **24**. The last approach can be considered as a synthetic way to efficiently prepare compounds of type **24** bearing the same aryls in the phosphonate and phosphate parts. If the aryl substituents are different, the synthesis of intermediates **5** and **22** has to be done separately and after putting them together, the addition of a tertiary amine triggers their coupling into *gem*-bisphosphonate **23** and the final phosphonate-phosphate **24**. In this approach special care is recommended since adventitious water can compromise the reaction. While the chemistry of phosphonate-phosphates is certainly very exciting, antiviral activities of compounds of type **24** were rather disappointing. The only exception was bisAZT phosphonate-phosphate **24** with Ar = 4-methoxy phenyl and Ar^1^ = phenyl that showed considerably high (EC_50_ = 0.01 μM) anti-HIV potency (Szymanska et al., 2006). Unfortunately, its pronucleotidic mode of action was not confirmed because the analogous ddU phosphonate-phosphate (Ar^1^ = phenyl, Ar = 4-methoxyphenyl) was not active at all against HIV. It is likely that the lack of antiviral activity of the studied phosphonate-phosphates was relevant to their breakdown in RPMI/FCS and depended on the electronic character of the R, Ar, and Ar^1^ substituents. Thus, compounds **24** with R = *t*-butyl or Ar = phenyl and Ar^1^ = 4-chlorophenyl were hydrolyzed into the respective unprotected dinucleoside phosphonate-phosphates, and appeared very resistant to further chemical or enzyme assisted hydrolysis. A quite different metabolism in cell culture media was observed for compounds **24** bearing Ar = 4-methoxyphenyl and Ar^1^ = phenyl that produced an equimolar mixture of nucleoside H-phosphonate **4** and aryl nucleoside phosphate diester of type **12**. The formation of phosphodiester **12** can be easily rationalized, whereas H-phosphonate **4** appeared to be the product of a more complex transformation via α-hydroxyphosphonate. It is likely that electron donating substituents (e.g., 4-methoxyphenyl) on the central carbon atom in **24** can stabilize carbocation after C-O bond scission and by weakening this bond, favors the departure of phosphate **12**. It might be postulated that less electron releasing R or Ar and more electron attracting Ar^1^ in dinucleoside phosphonate-phosphates **24** promote the hydrolysis of phosphoester bonds, while for the electron donating R or Ar, the formation of H-phosphonate **4** and phosphodiester **12** prevails in the hydrolysis of compounds **24** (Szymczak et al., 2003).

#### Nucleoside α-Hydroxyphosphonates

**Scheme 10:**
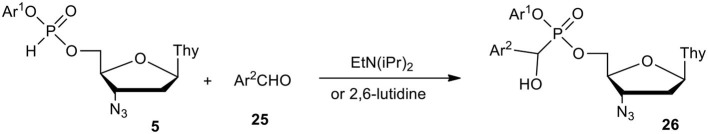
Scheme for the synthesis of aryl nucleoside α-aryl-α-hydroxyphosphonates of type **26** on the example of AZT derivatives.

Taking into account the putative involvement of α-hydroxyphosphonates as metabolites of phosphonate-phosphates in biological media, we investigated their synthesis and chemical properties in more detail, having in mind their impact on antiviral activity. Meier (1993); Meier et al. (1995a,b) showed that dinucleoside α-hydroxyphosphonates can be obtained in a reaction of dinucleoside H-phosphonate and aldehyde in the presence of an excess of tertiary amine (Et_3_N or Et_2_iPrN) at an elevated temperature. It was doubtful that the above conditions would be applicable to aryl nucleoside H-phosphonate of type **5** and milder chemistry was required for the synthesis of the respective α-hydroxyphosphonates of type **26** (Scheme 10). To this end, pre-generated aryl esters **5** (vide supra) were reacted with aromatic aldehyde **25** in the presence of a slight excess of Et_2_iPrN (for less electron attracting Ar^2^) or with less basic 2,6-lutidine (for strong electron attracting Ar^2^ in **25**). The use of 2,6-lutidine instead of Et_2_iPrN, although slowed down the addition of aldehyde **25** to ternalized phosphonate **5**, suppressed the formation of side products to an undetectable level (^31^P NMR). After purification by silica gel column chromatography, the designed nucleoside α-aryl-α-hydroxy-C-phosphonates of type **26** were obtained with yields exceeding 70% (Szymanska et al., 2006).

**Scheme 11:**
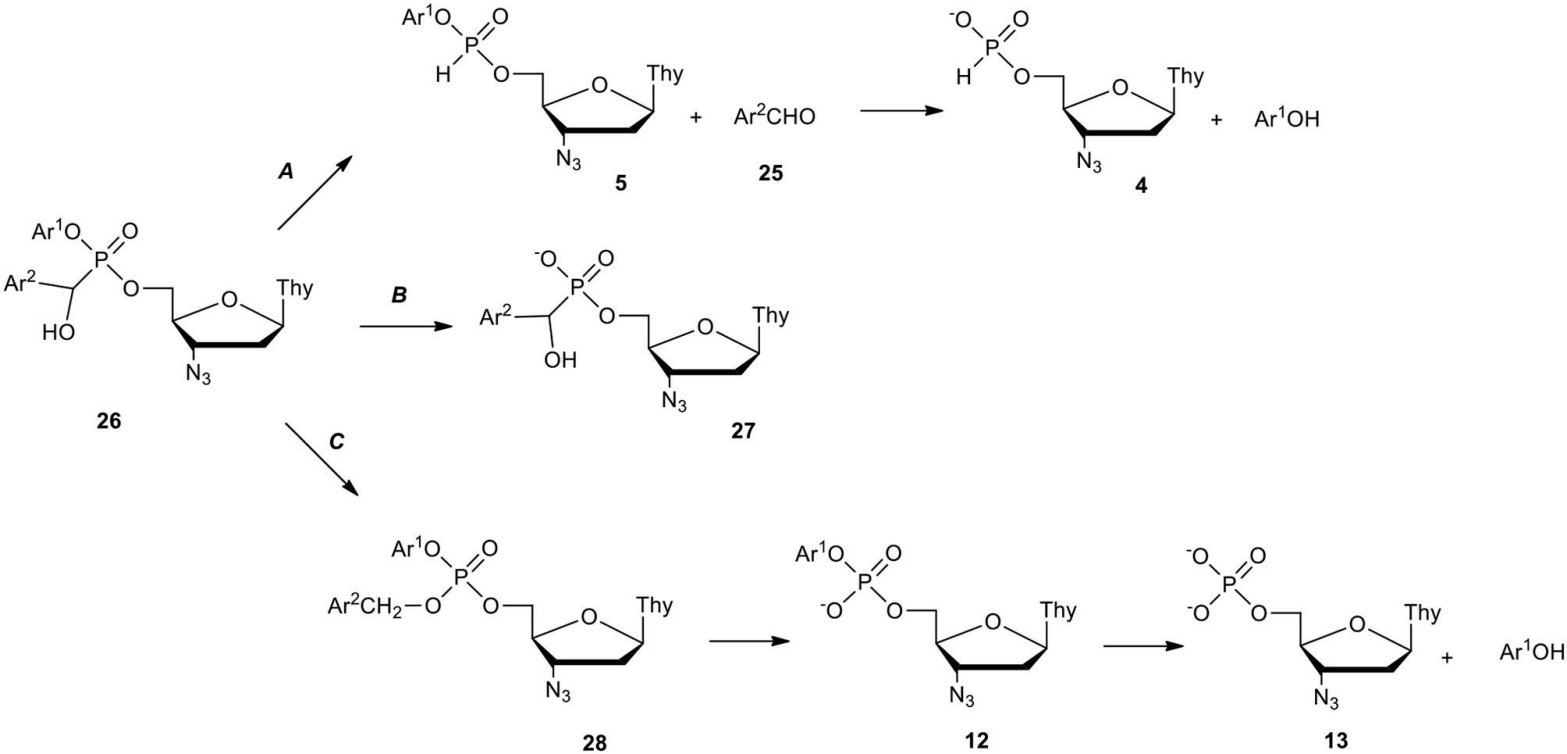
Decomposition paths of aryl nucleoside α-aryl-α-hydroxyphosphonates of type **26** in RPMI/FCS on the example of AZT derivatives.

Aryl nucleoside α-aryl-α-hydroxyphosphonates of type **26** delivered a lot of data during examination of their decomposition in cell culture media (RPMI/FCS). The degradation path of compound **26** strongly depended on the electronic and structural properties of Ar^1^ vs. Ar^2^ and on the basis of the structure of the degradation products formed. Three metabolic paths ***A***, ***B***, and ***C*** in RPMI/FCS were established (Scheme 11) (Szymanska et al., 2006). Path ***A*** which led to nucleoside H-phosphonate **4** was exclusive when both Ar^1^ and Ar^2^ had an electron donating character (Ar^1^ = phenyl, Ar^2^ = phenyl, 4-methylphenyl, 4-methoxyphenyl). It was proven that aryl nucleoside α-aryl-α-hydroxyphosphonates of type **26** equipped with electron donating aryls exist in equilibrium with aryl nucleoside H-phosphonates **5** and a respective aldehyde **25** which under hydrolytic conditions produced H-phosphonate **4** and phenol. Aryl groups Ar^2^ were able to donate electrons to stabilize hydroxybenzyl carbocation and promote the P-C bond scission favoring the formation of aldehyde **25** and H-phosphonate diester **5** which hydrolyzed toward the final H-phosphonate monoester **4**.

In contrast to this, the presence of electron withdrawing Ar^2^ (pyridin-3-yl, 4-nitrophenyl) at the α-carbon atom in compound **26** strengthened the P-C bond and favored decomposition path ***B***, in which aryl phosphoester bond hydrolysis occurred forming anionic α-aryl-α-hydroxyphosphonates **27** that were stable for a long time under the experiment conditions. 4-Nitrophenyl Ar^2^, the most electron withdrawing group in phosphonate **26** turned onto the third decomposition path ***C*** as a result of intramolecular self-oxidation and formation of phosphotriester of type **28**. The elimination of the 4-Nitrobenzyl group (also enhanced by Ar^1^ = 4-nitrophenyl) led to aryl phosphodiester **12** which in enzyme-assisted hydrolysis formed nucleoside phosphate **13** as the final product of the transformation of the phosphorus atom (Szymanska-Michalak et al., 2010).

Anti-HIV potency of a series of the investigated aryl AZT α-aryl-α-hydroxyphosphonates of type **27** was considerable, all in the range of EC_50_ value 0.01–0.06 μM (Szymanska et al., 2006) which was surprising in the light of their different metabolic paths and products. Compounds that decomposed according to path ***A*** exclusively (Ar^1^ = phenyl, Ar^2^ = phenyl, 4-methylphenyl, and 4-methoxyphenyl) disclosed similar anti-HIV activity as those with high involvement (> 40%) of path ***B*** (Ar^1^ = phenyl, Ar^2^ = 4-nitrophenyl or pyridin-3-yl) and at the same time were similarly cytotoxic (30–50 μM) (Szymanska et al., 2006). Thus, it can be presumed that anti-HIV potency of the investigated compounds **27** brought the same metabolite. The tested ddU analog (Ar^1^ = phenyl; Ar^2^ = 4-methoxybphenyl) disclosed no antiviral activity as well as cytotoxicity, which strongly suggested that the intermediate aryl AZT α-aryl-α-hydroxyphosphonates of type **27** were probably nucleoside vehicles and did not act as pronucleotides.

Considering the above it might be tentatively concluded that aryl nucleoside α-aryl-α-hydroxyphosphonates of type **27**, delivering very rich and unique products in some pointed chemistry, are not attractive compounds for pronucleotide strategy in antiviral therapy.

## Charged Antiviral Pro-nucleotides

### Anionic Antiviral Nucleoside (*N*-aryl)Phosphoramidates

**Scheme 12:**
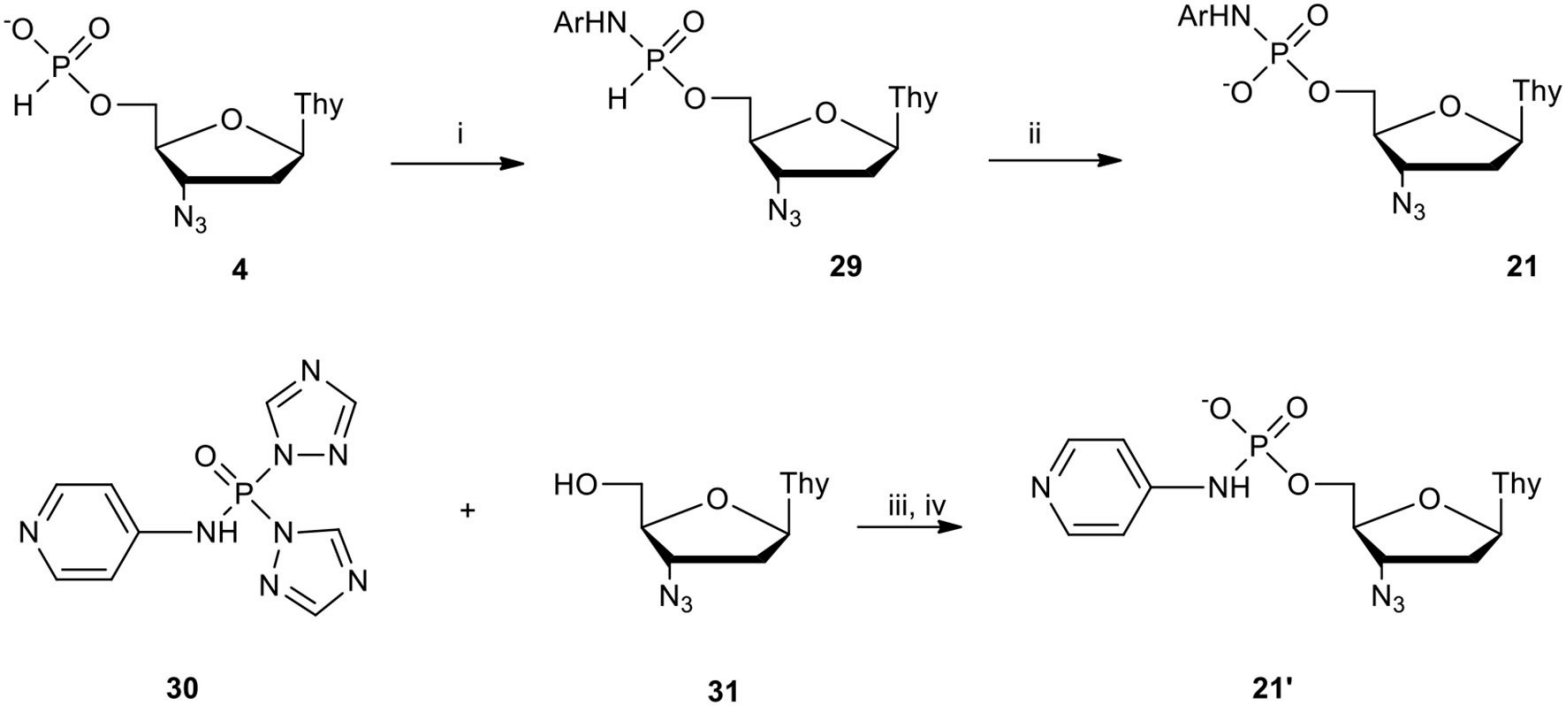
Scheme for the synthesis of nucleoside (*N*-aryl)phosphoramidates **21** and **21′** on the example of AZT derivatives. (i) **4** 1 molar equiv., ArNH_2_ 1.5 molar equiv., DPCP 1.5 molar equiv., in DCM/pyridine 9: 1 (v/v); (ii) I_2_ 2 molar equiv., pyridine containing H_2_O, 50 molar equiv.; (iii) **31** 2 molar equiv., **32** 1 molar equiv., 75°C, 10 min; (iv) H_2_O excess, 1 h.

With our experience and knowledge on the role of aryls masking groups in pronucleotides (vide supra), and inspired the discovery of C. R. Wagner (McIntee et al., 1997) that anionic amino acid-derived nucleoside phosphoramidates can enter cells and act as a progenitor of nucleotides, we focused our attention on (*N*-aryl)phosphoramidates of type **21** (Scheme 12) expecting to also find antiviral activity in this instance.

Compounds **29** bearing an Ar-NH-P bond differ essentially from their alkyl congeners in the synthetic context because most of the investigated aryl amines used in slight excess (1.5 molar equiv.) reacted easily with nucleoside H-phosphonate of type **4** activated with DPCP and produced the respective (*N*-aryl)phosphoramidates of type **29** quantitatively (Romanowska et al., 2011). Compounds **29** easily identifiable with ^31^P NMR spectroscopy (typical chemical shift of compounds of type **29** is in between 7.5 and 8.5 ppm and ^1^*J*_H−P_ ca. 650 Hz) (Romanowska et al., 2011) could not be isolated due to their instability and were subjected *in situ* to oxidation with the I_2_/H_2_O system (Garegg et al., 1987). The oxidation invariably proceeded smoothly [in contrast to (*N*-alkyl)-H-phosphonamidates of type **17**, vide supra] and products **21** after a simple workup were purified by silica gel column chromatography (yields over 70% in most of cases). The exception was 4-aminopyridine (Ar = pyridin-4-yl) that in a coupling reaction with H-phosphonate **4** generated a complex mixture of products. This was attributed to the involvement of the endocyclic nitrogen atom of 4-aminopyridine in the activation of H-phosphonate **4**. AZT (*N*-pyridin-4-yl)]phosphoramidate **21′** was thus obtained using a different approach, that is by phosphorylation of AZT **31** with *N*-bis(1*H*-1,2,4-triazol-1-yl)[(*N*-pyridin-4-yl)phosphoramidate (Romanowska et al., 2011) **30** (Scheme 12) which was developed for this purpose, obtained from phosphoryl tris-(1*H*-1,2,4-triazole) (Kraszewski and Stawinski, 1980) and equimolar amounts of 4-aminopyridine (Romanowska et al., 2011). Reagent **30** in pyridine at 75°C furnished phosphorylation of AZT within 10 min. The reaction was clean and the potential symmetrical products were not formed and the intermediate triazolo-phosphate (not shown) was hydrolyzed with an excess of water keeping the *N*-(4-pyridinyl) P-N bond untouched. With the exceptions of AZT phosphoramidate **21′** a series of nucleoside (*N*-aryl)phosphoramidates (Ar = phenyl, 4-methylphenyl, 4-chlorophenyl, pyridin-2-yl, pyridin-3-yl) of type **21** were obtained by a two-step one-pot procedure that can be considered as the general method for the synthesis of this type of compound.

The stability of nucleoside (*N*-aryl)phosphoramidates of type **21** examined in cell culture media showed that those bearing a pyridine-X-yl Ar substituent were much less stable when compared to simple phenyl and its 4-methyl- or 4-chloro derivatives. As the later ones were stable in the above conditions for over 5 days, *t*_1/2_ of pyridin-2 or 3-yl analogs were in the range of several dozen hours (Romanowska et al., 2011). To explain this we postulated and partially proved the existence of (*N*-pyridin-X-yl)phosphoramidates of type **21** in an equilibrium of charged (including zwitter-ions) and neutral species including those with protonated nitrogen atoms in the bridging P-N bond (Romanowska et al., 2011) which might facilitate its hydrolysis.

Among the examined nucleoside (*N*-aryl)phosphoramidates of type **21**, *N*-pyridinyl derivatives disclosed outstanding anti-HIV parameters (entries 1–3, Table 1). Besides very high antiviral potency (in nM range), their cytotoxicity was below the observable level even up to a concentration of 200 μM. As a result, exceptionally high selectivity indices (SI_50_ = CC_50_/EC_50_) were also generated pointing to these compounds as very good candidates for drug development in anti-HIV therapy. Their pronucleotidic feature was confirmed with antiviral activity disclosed by ddU analog which was also non-toxic showed considerably anti-HIV activity (Table 1, entry 4). It is important to note at this point that due to their good solubility in cell culture media these compounds could be tested in a wide range of concentrations which would certainly expand our knowledge on their anti-HIV potency.

**Table 1:** ^a^Stability of selected aryl AZT (*N*-aryl)phosphoramidates of type **21** in cell culture media, their cytotoxicity (CC_50_) against MT-4 cells and anti-HIV activity (EC_50_).

	**Nucleoside**	**Ar**	**RPMI t_**1/2**_ (h)**	**C**_**max**_ **(μM)^b^**	**CC_**50**_ (μM)**	**EC_**50**_ (μM)**	**SI_**50**_**
1	AZT	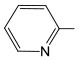	116.7	200	n. o.^c^	0.00241	>>83,500
2	AZT	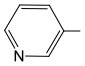	77.3	200	n. o.	0.0023	>>87,000
3	AZT	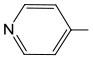	>5 days	200	n. o.	0.0011	>>182,000
4	ddU	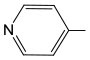	32.1 h	50	n. o.	0.2	>>250

a− *Abbreviations for CC_50_ and EC_50_ see footnote in the Introduction section*.

b− *Maximal concentration used in the assay*.

c− *n. o., not observed*.

To find the origin of very high anti-HIV potency of AZT (*N*-pyridin-X-yl)phosphoramidates **21** we considered P. Herdewijn's (Adelfinskaya and Herdewijn, 2007) scenario in which conjugates of deoxynucleotides and amino acids were found to be alternative substrates for HIV-RT polymerase. To this end using an *in vitro* system of HIV-RT polymerase, the inhibitory ability of compounds **21** was tested. Unfortunately none of them showed a measurable inhibition of HIV-RT in concentrations up to 3 μM (Romanowska et al., 2011). The other possibility could be that (*N*-pyridin-X-yl)phosphoramidates of type **21** were converted with cellular phosphoramidases into respective nucleoside phosphates and further into triphosphates, inhibitors of cDNA synthesis by HIV-RT polymerase. Since compounds of type **21** appeared resistant to hydrolysis (Romanowska et al., 2011) when treated with Fhit (Guranowski et al., 2008) or Hint (Guranowski et al., 2010a,b) proteins, it is doubtful that the examined compounds could be substrates of this type of enzyme, unless in the cells exists a so far unknown phosphoramidase able to hydrolyze the P-N bond of the investigated phosphoramidate nucleotide analogs and turns them into substrates for the synthesis of the corresponding nucleoside triphosphate, inhibitors of viral cDNA synthesis. Despite of lack of evidence supporting the mode of action at the molecular level of AZT (*N*-pyridin-X-yl)phosphoramidates of type **21** as anti-HIV agents, their excellent therapeutic parameters allowed their patenting in Europe (Kraszewski et al., 2014) and USA (Kraszewski et al., 2015) as a potential drug in AIDS therapy.

### Anticancer Zwitter-Ionic Pro-nucleotides

**Scheme 13:**
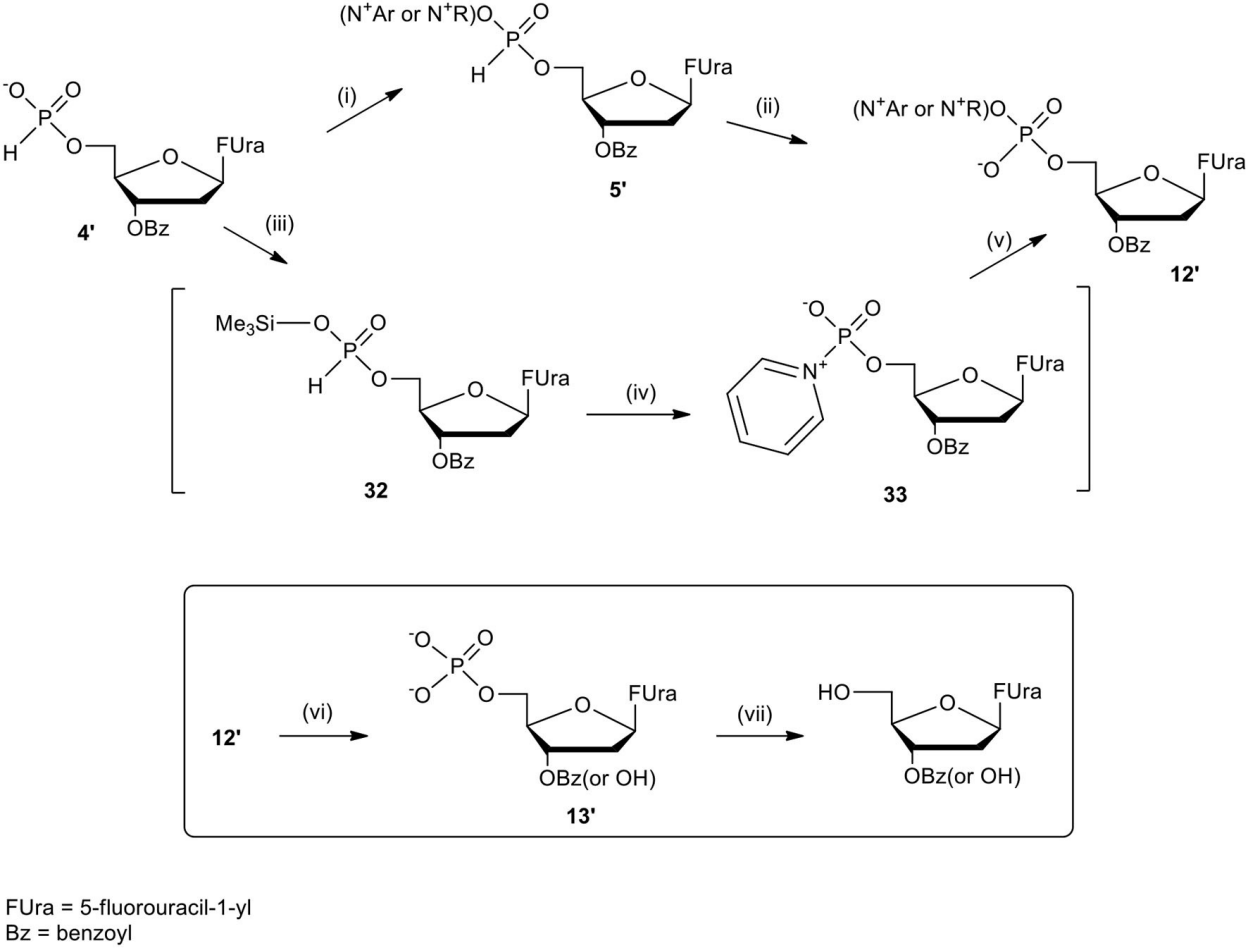
Synthesis of FdUMP X-aminium phosphodiesters of type **12′** and their decay (inset) in RPMI/FCS 9: 1 (v/v), 37°C. (i) 1 mmol of **4′** in 10 mL of CH_2_Cl_2_/pyridine 9: 1 v/v, ArOH or ROH 1.5 mmol, 1.5 mmol diphenylchlorophosphate, 5 min, r. t.; (ii) I_2_ 2 mmol in 5 mL of pyridine, after 10 s, H_2_O > 5 min; (iii) **4′**, 1 mmol in acetonitrile, 2 mmol TMSCl and Et_3_N; ArOH 2.5 mmol; (iv) 2 mmol of I_2_ in pyridine < 3 min; (v) 2.5 mM of ArOH; (vi) FCS phosphoesterase activity; and (vii) FCS phosphatase activity.

The last concept of our pronucleotides reconciles the presence of a charge and lipophilicity in a form of zwitter-ionic electrically neutral molecules bearing one or more lipophilic substituents in the nucleoside or in the phosphate residue. In our laboratory, the first target for zwitter-ionic pronucleotides were glioblastoma cells that are known to overexpress thymidylate synthase (TS) (Grunda et al., 2006), and thus as a nucleoside part 2′-deoxy-5-fluorouridine (FdU) was chosen since its 5′-phosphate is an effective inhibitor of this enzyme (Hartmann and Heidelberger, 1961). The general idea of our antiglioma pronucleotides was presented on the example of compound **12′** (Scheme 13) in which besides 3′-O-protected nucleoside there is a phosphate part in the form of zwitter-ionic betaine. On the grounds of our earlier studies, we chose 3′-*O*-benzoyl-2′-deoxy-5-fluorouridine as a nucleoside part (Szymanska-Michalak et al., 2016) and phosphoesters of various *N*-alkylpyridinols or (*N-*tetra-substituted) amino-alcohols both positively charged at the nitrogen atom (Szymanska-Michalak et al., 2018). As an initial substrate, protected FdU H-phosphonate **4′** was used but early experiments showed that the synthesis of the target pronucleotide of type **12′** strongly depended on the hydroxyl component in the coupling reaction (Scheme 13).

For referential arylols (i.e., phenol, pyridinol-3, quinolinol-6) and all alkyl amino-alcohols [2-(*N,N,N-*trimethylaminium)ethanol, 2-(*N*-benzyl-*N,N*-dimethylaminium)ethanol, *N,N*-dimethylpiperidinol-4], the “classical” path via respective H-phosphonate diesters of type **5′** worked perfectly well. The coupling of nucleoside H-phosphonate **4′** with the above mentioned arylols or amino-alcohols in CH_2_Cl_2_/pyridine 9: 1 (v/v) aided with DPCP uneventfully produced H-phosphonate dieters **5′** which after oxidation with I_2_ in a pyridine-water mixture furnished the awaited nucleoside phosphodiester **12′**. Unfortunately, this synthetic route was abandoned because of the similar coupling of H-phosphonate **4′** with *N*-alkyl pyridinols [*N*-methylpyridinol-3, *N*-decylpyridinol-3, *N*-benzylpyridinol-3, *N*-(carboetoxymethyl)pyridinol-3] was unsuccessful most likely due to the similar nucleophilicity of the pyridinols and other nucleophiles present in the coupling reaction. Another route was developed that proceeded with the intermediacy of nucleoside pyridinium-1-ylphosphonate **33** (Scheme 13). To this end nucleoside H-phosphonate **4′** was silylated with trimethylsilyl chloride in pyridine toward silyl ester **32** which in a reaction with I_2_ in the presence of pyridine furnished pyridinium-1-ylphosphonate **33** as the main compound, which due to its instability could not be isolated but could be well-characterized with ^31^P NMR spectroscopy and chemically. Compound **33** in reactions with a slight excess of the aforementioned “unreactive” *N-*alkyl pyridinols smoothly produced the expected zwitter-ionic nucleotides **12′**. The route with intermediacy of reactive pyridinium derivative **33** could be executed in a one-pot procedure what ensured a high yield of products, and can be considered as a convenient and more general alternative for other synthetic methods.

In order to evaluate the potential applicability of the obtained zwitterionic FdU pronucleotides of type **12′** as cytostatic drugs, their paths of decay in RPMI/FCS were studied. It was found that all the investigated compounds in cell culture media decomposed similarly with a *t*_1/2_ between 6 and 20 h, and produced the awaiting nucleotide **13′** (Scheme 13, inset) with unshielded phosphate residue and also with a partially hydrolyzed 3′-*O*-benzoil group. Both compounds when kept for a longer time (*t*_1/2_ > 20 h) in cell culture media lost their phosphate residue because of FCS phosphatase activity. Nevertheless, in the first several hours of the experiment, besides zwitter-ionic substrate **12′**, the main product observed was FdU phosphate **13′** and it could be expected that in the cell this compound would dominate as the metabolite and trigger the expected biological effects (Szymanska-Michalak et al., 2018).

Opposite to antiviral nucleotidic drugs, anticancer pronucleotides should destroy cancer cells preferentially, leaving healthy ones unharmed. The studied glioma cell lines T98G, U-119 MG, and U-87 MG were treated with FdU pronucleotides of type **12′** and as a reference, healthy cell line MRC-5 was used (Szymanska-Michalak et al., 2018). In the case of anticancer prodrugs, besides cytotoxicity (IC_50_) which describes the concentration of the compound that inhibits in 50% cell viability/proliferation, the most important measurement is selectivity index (SI), i.e., the quotient of IC_50_ of healthy cells and IC_50_ of cancer cells. The last parameter describes the real therapeutic anticancer value of an investigated compound - the higher the value, the more selective the compound is.

In standard anti-proliferative tests (Xavier et al., 2011) all the compounds investigated disclosed a pronounced activity in a wide range of μM concentrations (0.53–66.28 μM) against glioma cell lines T98G, U-118 MG, and U-87 MG, and were at the same time less harmful toward referential healthy lung fibroblast MRC-5 cells, referential HeLa cancer cell lines, and Caco-2 cells used as mimics of intestine tissue (Szymanska-Michalak et al., 2018). However, among the examined compounds those bearing zwitter-ionic aryl masking functionality (Table 2, entry 3–6) at the phosphate part, exhibited outstanding anti-proliferative selectivity in comparison to neutral aryl phosphoesters (vide phenyl, Table 2, entry 1) or zwitter-ionic alkyl ones. The same tendency could be observed for simple pyridine-3-yl aryl in **12′** (Table 2, entry 2) that most likely can exist also as zwitter-ion, analogously to its phosphoramidate congener (Romanowska et al., 2011) (vide supra). It could be suspected that zwitter-ionic species might easier penetrate cancer cell membranes than other type of cells and cause their death [analogously to membrane interactive anticancer drugs miltefosine (Dorlo et al., 2012) or perifosine (Gills and Dennis, 2009)]. It is worth stressing that the anti proliferative potency of the examined compounds **12′** strongly depended on the glioma cell line, and thus their therapeutic value cannot be generalized on the basis of data obtained from experiments on one cell line. Nevertheless, the investigated herein *N*-substituted pyridinium zwitter-ion nucleotides of type **12′** were found to be selectively active against glioma cells and are certainly worthy of further investigation.

**Table 2:** SI_50_ values for zwitter-ionic 3′-*O*-benzoyl-2′-deoxy-5-fluorouridine pronucleotides of type **12′** that disclosed the most convenient anticancer parameters.

**Entry**	**Cpd 12^**′**^**	**IC_**50**_ (μM)**	**SI**_****50****_		
	**Ar**	**MRC-5**	**T98G**	**U-118 MG**	**U-87 MG**
1	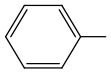	1.27	2	1	2
2	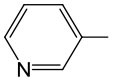	15.06	12	17	22
3	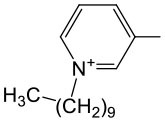	14.01	35	44	21
4	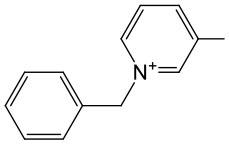	38.5	15	26	58
5	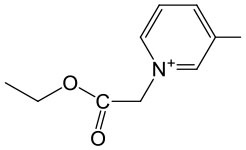	8.60	5	6	15
6	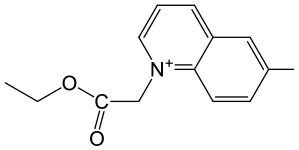	34.9	3	46	66

## Summary

Pronucleotides, besides being therapeutic (usually nucleoside analog), have to be equipped with structural elements that by means of proper interactions allow them to cross respective membrane [e.g., cell membrane or brain blood barrier (BBB)] and reach their target. There are many examples of compounds which in cell free systems are very active while being useless in cellular or organismal therapy because they are not able to penetrate the protective membranes mentioned above. Among nucleoside drugs, several of them can cross cellular membranes in spontaneous diffusion (e.g., AZT), and others with nucleoside transport systems (Zhu et al., 2017). In pronucleotides, a phosphate part has to be decorated with appropriate functionalities to mask phosphate negative charge otherwise these compounds cannot be internalized by cells efficiently (Remy et al., 1962). Thus, the chemical challenge was to find an optimal system for masking phosphate residue that could meet several, sometimes opposite requirements, for instance lipophilicity vs. solubility in an aqueous environment. One of the most important aspects concerning pronucleotides is their biocompatibility with cellular physiological machinery that in most cases is indispensable for the conversion of a decorated pronucleotide into its biologically active form. Described herein H-phosphonate chemistry which was developed or adopted for the preparation of a variety of pronucleotides fulfills these needs to a large extent. Most (if not all) phosphate modified pronucleotides were obtained with high efficiency and under mild conditions (room temp., slightly basic conditions, volatile organic solvents) which opened the access to a variety of compounds bearing fragile structural element(s) (e.g., nucleoside analogs or decomposition-prone functional groups). This we exploited in the preparation of uncharged diaryl nucleoside phosphotriesters, aryl nucleoside (*N*-aryl)phosphoramidates, fully masked dinucleoside phosphonate-phosphates, nucleoside α-aryl-α-hydroxy)phosphonates, anionic nucleoside (*N*-aryl)phosphoramidates, and zwitter-ionic nucleoside phosphodiesters. In their synthesis aryl H-phosphonates diesters were are not stable enough to be isolated but were compounds of choice in several synthetic tasks because the activity of aryl H-phosphonate diester can be precisely tuned by the means of substituents in the aromatic system. Several types of pronucleotides were developed and many of them disclosed their antiviral or anticancer activity at μM or even nM levels. Among anti-HIV pronucleotides AZT (*N*-pyridin-X-yl)phosphoramidates showed an outstanding antiviral potency, they were highly active and practically non-toxic for infected MT-4 cells. In studies of anticancer pronucleotides we found FdU zwitter-ionic phosphodiesters to be selectively and highly active against glioma cell lines what certainly encourages their further development. It is worth adding that the last 2 years in our laboratory studies on the chemistry of boranephosphonates (Golebiewska et al., 2018; Golebiewska and Stawinski, 2020) may provide a valuable extension of our recent studies on pronucleotides containing a boranephosphonate skeleton (Li et al., 2007; Roy and Caruthers, 2013).

## Author Contributions

All authors listed have made a substantial, direct and intellectual contribution to the work, and approved it for publication.

## Conflict of Interest

The authors declare that the research was conducted in the absence of any commercial or financial relationships that could be construed as a potential conflict of interest.

## Footnotes

^1^EC_50_ - concentration required to achieve 50% protection cells against virus-induced cytopathogenicity (effective concentration). CC_50_ - concentration required to reduce the viability of mock-infected cells by 50% (cytotoxic concentration).
